# Supplementary material for: Risk factors for sacrococcygeal pilonidal sinus: a systematic review and meta-analysis supplemented by genetic causal assessment
Source: Front Surg. 2026 Jan 7;12:1718589. doi: 10.3389/fsurg.2025.1718589 (PMC12819706; doi:10.3389/fsurg.2025.1718589)
Supplement: Supplementary file 2 [file Datasheet2.zip › Supplementary Data 2/MR_pipeline_p5e-8/forest_plots/ukb-d-1747_3.finngen_R12_L12_PILONIDALCYST.pdf]

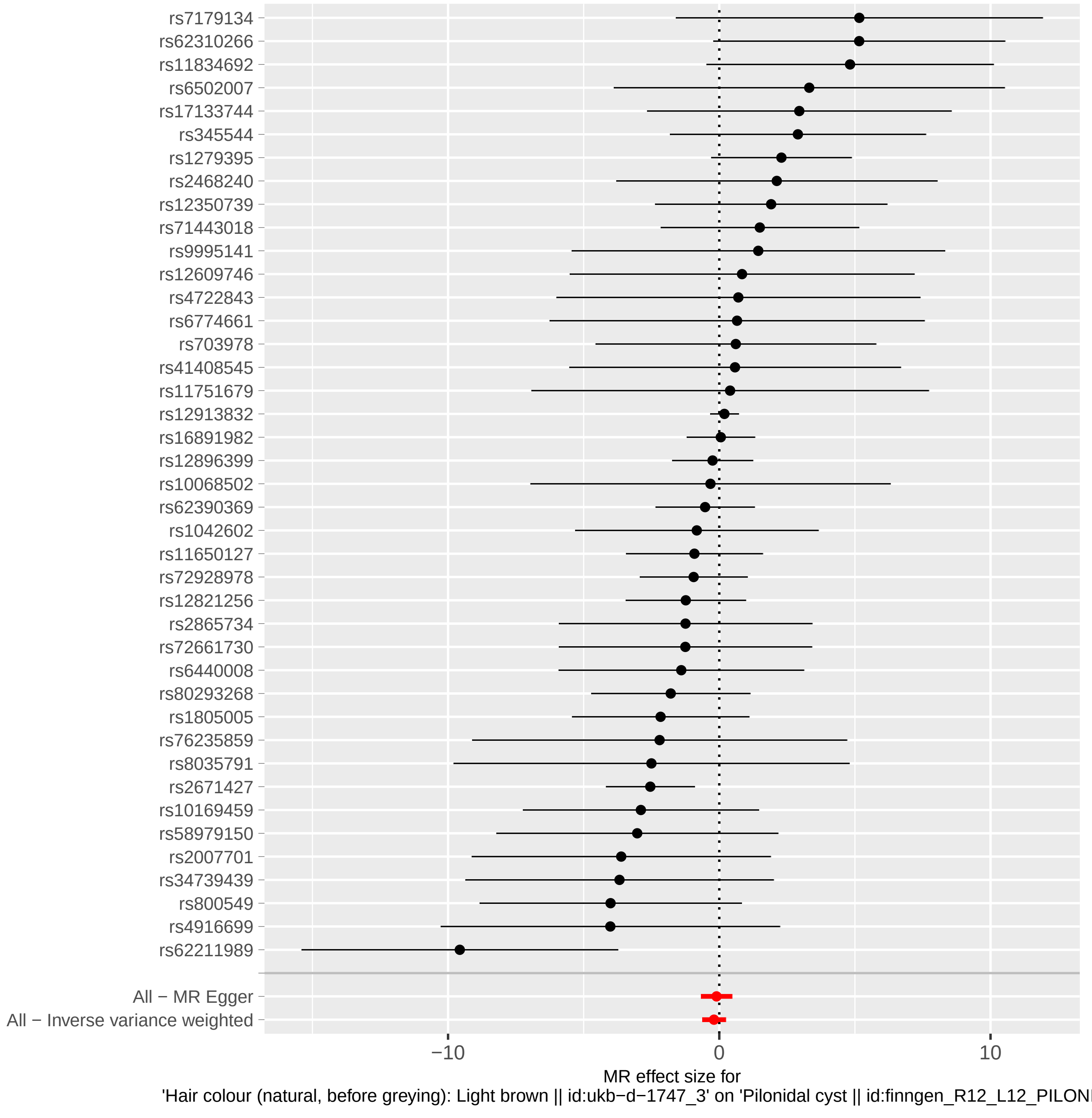
rs7179134
rs62310266
rs11834692
rs6502007
rs17133744
rs345544
rs1279395
rs2468240
rs12350739
rs71443018
rs9995141
rs12609746
rs4722843
rs6774661
rs703978
rs41408545
rs11751679
rs12913832
rs16891982
rs12896399
rs10068502
rs62390369
rs1042602
rs11650127
rs72928978
rs12821256
rs2865734
rs72661730
rs6440008
rs80293268
rs1805005
rs76235859
rs8035791
rs2671427
rs10169459
rs58979150
rs2007701
rs34739439
rs800549
rs4916699
rs62211989
All − MR Egger
All − Inverse variance weighted
−10
0
10
MR effect size for
'Hair colour (natural, before greying): Light brown || id:ukb−d−1747_3' on 'Pilonidal cyst || id:finngen_R12_L12_PILON
